# Cognitive load and pedagogical tension in multi-platform online learning: Evidence from Chinese higher education

**DOI:** 10.1371/journal.pone.0347566

**Published:** 2026-04-21

**Authors:** Xiaoqin Qiu, Nengsheng Qiu

**Affiliations:** 1 Department of General Education, Xiamen Medical College, Xiamen, Fujian, China; 2 School of Foreign Languages, Xiamen Institute of Technology, Xiamen, Fujian, China; PNG National Research Institute, PAPUA NEW GUINEA

## Abstract

The proliferation of digital tools has transformed higher education into a complex, multi-platform online learning environment. This study investigates the paradoxical effects of this multi-platform environment on the student experience within Chinese higher education. Through the qualitative and quantitative data from a cross-sectional survey of 8,616 university students, we analyzed platform usage patterns, perceived benefits and challenges. The findings reveal a significant paradox: while students express positive attitudes towards platforms’ educational value, acknowledging benefits like enhanced self-directed learning and improved teacher-student interaction, they concurrently report substantial practical burdens. A serial mediation model confirmed that platform multiplicity increases extraneous cognitive load, which in turn elevates tool fatigue, ultimately leading to a more negative perception of the learning experience. This study identifies a core “pedagogical tension” between the intended benefits of educational technology and the lived reality of a fragmented, high-friction user experience that encourages instrumental engagement over deep learning. These findings underscore the urgent need for institutions and instructors to adopt a more strategic, integrated approach to educational technology to reduce fragmentation and prioritize a seamless student learning experience.

## Introduction

The landscape of higher education has been irrevocably transformed by the integration of digital technologies. What began as a supplementary tool has evolved into a core component of the modern university experience. The COVID-19 pandemic acted as a powerful catalyst, compelling a near-universal and abrupt shift to online and blended learning models [1]. This transition not only normalized the use of technology in pedagogy but also accelerated the diversification of the digital tools employed. Universities and instructors, seeking to replicate the multifaceted nature of in-person instruction, moved beyond the confines of a single, centralized Learning Management System (LMS) like Moodle or Blackboard. They began adopting a suite of specialized applications for video conferencing, collaborative work, subject-specific simulations, and assessment, creating a complex, multi-platform digital ecosystem [2].

This proliferation of platforms presents a double-edged sword. On one hand, it offers unprecedented flexibility, resource accessibility, and the potential for innovative pedagogical approaches tailored to specific learning objectives. Platforms like Tencent Meeting or DingTalk facilitate synchronous communication, while specialized tools like I-WRITE or Pigai.org offer focused practice in specific skills. This “best-of-breed” approach allows instructors to select the optimal tool for each task [3].

On the other hand, this fragmentation introduces significant challenges for the end-users: the students. Navigating a multitude of platforms, each with its own unique interface, login credentials, notification system, and workflow, can impose a substantial cognitive and administrative burden. This phenomenon, often termed “tool fatigue” or “platform proliferation” [4], risks shifting the student’s focus from learning the course content to managing the technology required to access it. The potential for a disjointed learning experience, where communication, resources, and assessments are scattered across disparate and non-integrated systems, is a growing concern for educators and institutions alike.

This issue is particularly salient in the context of Chinese higher education, which is characterized by its immense scale and rapid adoption of domestic technology. The survey data for this study, gathered from over 8,600 students, indicates the dominance of local platforms such as Xuexitong, Tencent Meeting, and DingTalk. Understanding the student experience within this specific technological and cultural context is crucial for developing effective digital education strategies.

While extensive research has explored the efficacy of online learning and the role of single LMSs, there remains a gap in the literature concerning the cumulative effect of using multiple platforms on student learning, engagement, and well-being. This paper aims to address this gap by analyzing a large-scale survey of Chinese university students. It seeks to move beyond a simple evaluation of individual platforms to understand the holistic experience of navigating a complex digital learning environment. The central research questions guiding this study are:

1. *What is the prevalence and nature of multiple online learning platform usage among Chinese college students?*
2. *What are the primary purposes for which students use these platforms?*
3. *What are the perceived benefits of using these platforms for their learning and skill development?*
4. *What are the significant drawbacks and challenges students encounter when navigating this multi-platform environment?*
5. *How do students ultimately perceive the overall impact of this multi-platform usage on their educational experience?*

By systematically analyzing the survey data, this paper will first present the landscape of platform usage, then delve into the perceived benefits and documented challenges, and finally discuss the critical tension between these two aspects. The paper will conclude with implications for key stakeholders—institutions, instructors, and technology developers—arguing for a more strategic and integrated approach to educational technology adoption.

## Literature review

The integration of technology into higher education is not a new phenomenon, but its character and intensity have evolved dramatically. This review will trace this evolution, establish the theoretical frameworks used to understand its impact, and situate the current study within the existing body of knowledge, highlighting the specific research gap it aims to fill.

### The evolution from single LMS to a multi-platform ecosystem

For decades, the primary model for digital learning in higher education was the centralized Learning Management System (LMS). Systems like Blackboard, Moodle, and Canvas provided a “walled garden” environment, a one-stop-shop for course materials, gradebooks, discussion forums, and assignment submissions [5]. The primary advantage of the LMS was its integration and consistency, providing a predictable and unified experience for students and faculty across an institution.

However, the monolithic nature of the traditional LMS often led to criticism of it being a “content repository” rather than a dynamic learning environment [6]. The global pivot to emergency remote teaching during the COVID-19 pandemic exposed the limitations of relying solely on an LMS. Instructors and students rapidly adopted a wider array of tools to meet specific needs: Zoom and Tencent Meeting for synchronous video, Slack and DingTalk for real-time communication, and a host of other collaborative and specialized applications [7]. This has led to the emergence of the “Next Generation Digital Learning Environment” (NGDLE), a concept that envisions a decentralized, interoperable ecosystem of tools rather than a single system [3]. While this model promises greater flexibility and innovation, it is the very source of the platform proliferation problem this study investigates.

### Theoretical frameworks for understanding technology in learning

Several theoretical lenses are useful for analyzing the impact of this multi-platform environment on students. Cognitive Load Theory (CLT), developed by John Sweller, posits that working memory is limited and that effective instructional design must manage the cognitive load imposed on learners. Load is categorized into intrinsic, germane, and extraneous components [8]. In a multi-platform environment, students must constantly switch between different user interfaces, remember multiple login credentials, and track assignments across various systems. These tasks increase extraneous cognitive load, reducing the cognitive resources available for deep learning [9].

The Technology Acceptance Model (TAM), proposed by Davis, suggests that two key factors determine a user’s intention to use a system: Perceived Usefulness and Perceived Ease of Use [10]. In mandatory educational settings, Perceived Usefulness is often high by default, but the proliferation of platforms can severely diminish the Perceived Ease of Use, leading to frustration and negative attitudes, even if the technology is ultimately adopted out of necessity.

Community of Inquiry (CoI) Framework, developed by Garrison, Anderson, and Archer, emphasizes cognitive, social, and teaching presence as foundations for meaningful online learning [11]. While multiple platforms may offer more channels for interaction (e.g., video, chat, forums), the fragmentation of these channels can hinder the development of a cohesive community and a unified teaching presence, potentially compromising all three elements of the CoI framework.

**Fig 1:**
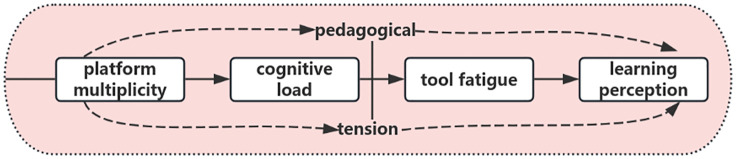
A conceptual framework of platform multiplicity’s impact on learning perception via cognitive load and tool fatigue.

The present study integrates these frameworks into a conceptual model (Fig 1) explaining how platform multiplicity shapes cognitive load, tool fatigue, and ultimately students’ learning perceptions.

In this framework, platform multiplicity is theorized to increase extraneous cognitive load by requiring students to navigate multiple interfaces, manage redundant tasks, and coordinate dispersed learning activities. Elevated extraneous load contributes to tool fatigue, a form of technology‑related exhaustion that arises from repeated interactions with fragmented digital tools. Tool fatigue, in turn, shapes students’ learning perception, influencing their sense of coherence, ease, and overall satisfaction with the learning experience. Pedagogical tension functions as an overarching experiential condition throughout this pathway, reflecting the perceived misalignment between instructional intentions and the fragmented learning environment. This framework specifies testable mechanisms linking technological complexity to students’ cognitive and affective learning outcomes.

In this study, pedagogical tension is defined as a core psychological conflict experienced by students in fragmented digital learning environments. It refers to the dissonance between learners’ rational recognition of the pedagogical value of educational technologies—such as their potential to enhance learning outcomes or develop digital competencies—and their lived experiences of practical friction and cognitive burden arising from the simultaneous use of multiple, non-integrated platforms. This tension is not merely dissatisfaction with technology, but rather stems from a misalignment between well-intentioned instructional design and the emergent reality of a disjointed, high-effort user experience.

### Benefits and challenges of online platforms

The literature extensively documents the benefits of online learning platforms. They provide flexibility in time and place, offer access to a vast array of digital resources, and can facilitate collaborative learning and provide instant feedback [12]. Platforms can support a variety of pedagogical approaches, from constructivist projects to direct instruction, and the data they generate can offer insights into student progress.

Conversely, the challenges are equally well-documented. These include the digital divide (inequalities in access to technology and digital literacy), issues of student motivation and self-regulation, and a sense of isolation [13]. More specific to the multi-platform issue, researchers have begun to identify “technostress” and “tool fatigue” as significant negative factors. Technostress arises from the pressure to adapt to new technologies and the constant connectivity they demand [14]. Tool fatigue describes the exhaustion and decreased performance resulting from having to use too many digital tools [4]. This fragmentation can lead to information overload, missed communications, and a general sense of being overwhelmed [15].

### The Chinese context and the research gap

China’s higher education system, the largest in the world, has embraced “educational informatization” as a national strategy [16]. This has led to the development and widespread adoption of domestic platforms like Xuexitong (from the Chaoxing company), Tencent Classroom/Meeting, and DingTalk, which are tailored to the local educational and technological environment. The scale of this adoption makes China a critical case study for understanding the effects of platform-based learning.

While many studies have examined online learning in China, particularly after the pandemic (e.g., Bao, 2020), most have focused on the general shift to online modalities or the effectiveness of a single platform. Few have specifically investigated the student-level experience of navigating an ecosystem of multiple, often non-integrated, platforms. The current study addresses this gap by using a large dataset to quantify the extent of platform proliferation and analyze the resulting tension between the stated benefits and the practical challenges faced by students on a daily basis. It connects the practical problems reported by students (e.g., forgetting passwords, network crashes) to established theoretical frameworks like CLT and TAM to provide a deeper analysis of the underlying issues.

## Materials and methods

This study employs a quantitative, cross-sectional survey design to investigate the use of multiple online learning platforms among college students in China. This approach is well-suited for capturing a broad snapshot of behaviors, perceptions, and attitudes from a large and diverse population at a single point in time.

### Research instrument

The data for this analysis was drawn from a pre-existing survey titled *Survey on the Use of Online Course Learning Platforms by University Students*. The questionnaire was initially designed in Chinese and later translated into English. The survey instrument was a structured questionnaire consisting of 21 items, divided into several thematic sections:

Demographics (Questions 1–4): This section collected data on respondents’ gender, type of institution (985/211, regular undergraduate, vocational), academic major, and year of enrollment.

Platform Usage Patterns (Questions 5–7): These questions identified which platforms students used, the total number of platforms used for all courses, and the number of platforms used specifically for English courses.

Nature and Purpose of Use (Questions 8–12): This section explored whether platform use was mandatory, students’ perceptions of the quantity of platforms, the primary purposes for using them (e.g., attendance, assignments, interaction), the timing of use (in-class vs. out-of-class), and the types of interactive features engaged with.

Challenges and Benefits (Questions 13–14): These multiple-choice questions asked students to identify the problems and difficulties they encountered while using the platforms and to select the abilities or literacies they felt were enhanced through platform use.

Attitudinal Perceptions (Questions 15–20): This section used a Likert-type scale format (Strongly Agree, Basically Agree, Basically Disagree, Strongly Disagree, Unable to Determine) to gauge student agreement with statements about the platforms’ impact on teacher-student interaction, student-student interaction, personal learning habits, and overall educational effectiveness. Internal consistency was assessed for the six items measuring students’ perceptions of online learning platforms. Cronbach’s α for the combined scale was 0.934, indicating high reliability. As shown in Table 1, item–total correlations ranged from 0.771 to 0.855, and Cronbach’s α did not improve meaningfully with the deletion of any item (α if deleted ranged from 0.916 to 0.927). These results demonstrate that the six items form a coherent and internally consistent scale.

**Table 1:** Reliability analysis: Item-total statistics for attitudinal perceptions questions.

	Item-total statistics			
	Scale mean if item deleted	Scale variance if item deleted	Corrected item-total correlation	Cronbach’s alpha if item deleted
Q15	11.19	22.380	.776	.926
Q16	11.15	22.064	.784	.925
Q17	11.27	22.473	.810	.922
Q18	11.24	21.976	.855	.916
Q19	11.27	22.202	.844	.918
Q20	11.22	22.555	.771	.927

Qualitative Feedback (Question 21): The final item was an open-ended question asking for keywords related to students’ evaluation and reflection on their platform use. It provides a deeper, more nuanced understanding of the reasons behind the quantitative responses as the keywords provided by students could offer valuable context for their satisfaction and frustrations.

### Participants and data collection

The dataset comprised responses from 8,616 university students from various higher education institutions in China. The data was collected through an online survey which lasted from February 28, 2023 to June 19, 2024.

**Fig 2:**
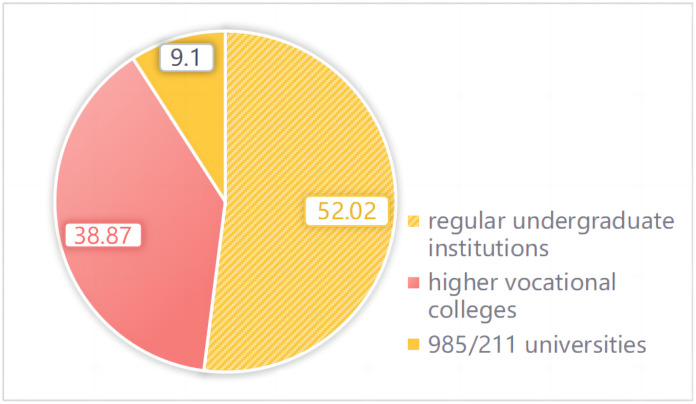
Institution type of respondents.

**Fig 3:**
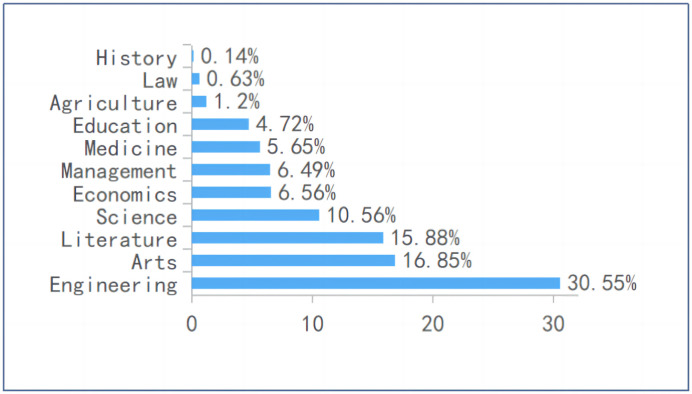
Academic major of respondents.

The demographic profile of the respondents revealed a diverse representation in terms of gender, institution type, academic major, and year of enrollment. Among the participants, 55.05% identified as female and 44.95% as male. The majority of students were enrolled in regular undergraduate institutions (52.02%), followed by higher vocational colleges (38.87%) and a smaller portion from elite 985/211 universities (9.11%) (see Fig 2). This distribution provides a comprehensive view of the tiers within Chinese higher education. In terms of academic majors, Engineering accounted for the largest group (30.55%), followed by Arts (16.85%), Literature (15.88%), and Science (10.56%) (see Fig 3), allowing for insights beyond specific disciplines. The sample was notably skewed towards newer students, with 66.84% being freshmen (enrolled in 2022) and 24.33% sophomores (enrolled in 2021), suggesting that their perceptions may reflect an early adaptation phase to university-level digital learning.

### Data analysis

The analysis was conducted using descriptive statistics. For all closed-ended questions (Questions 1–20), frequencies and percentages were calculated to summarize the responses. This method is appropriate for providing a clear and accurate overview of the students’ reported experiences and perceptions. The results were then organized thematically to align with the research questions, covering the landscape of platform use, the purposes of engagement, perceived benefits, and encountered challenges. The analysis focuses on identifying key trends, patterns, and notable contradictions within the data. For the Likert-scale questions, the “Strongly Agree” and “Basically Agree” categories were combined to represent an overall positive sentiment, while “Strongly Disagree” and “Basically Disagree” were combined to represent a negative sentiment, allowing for a clear interpretation of student attitudes.

### Ethical considerations

The study protocol involving human participants was reviewed and approved by the Ethics Review Committee of Xiamen Institute of Technology (Reference No: XITSLF202301, approved on January 6, 2023). The study was conducted in accordance with the Declaration of Helsinki and all relevant local legislation and institutional requirements. All participants were fully informed about the purpose and procedures of the research and provided their informed consent prior to their participation in this study.

## Findings

This section presents the results of the survey, organized into five key themes that directly address the research questions: the landscape of platform usage, the purpose and nature of student engagement, the perceived benefits and skill development, and the challenges encountered in the multi-platform environment.

A series of hierarchical regressions were conducted to test the serial mediation model. Platform multiplicity was positively associated with cognitive load (β = 0.062, p < .001). Cognitive load significantly predicted tool fatigue (β = 0.064, p < .001). Tool fatigue was negatively associated with learning perception (β = –0.771, p < .001). In the full model including both mediators (see Table 2), the direct effect of platform multiplicity on learning perception was non-significant (β = 0.005, p = .444), while cognitive load (β = 0.058, p < .001) and tool fatigue (β = –0.768, p < .001) remained significant. The model explained 59.8% of the variance in learning perception, F(3, 8612) = 4265.31, p < .001.

**Table 2:** Hierarchical regression model predicting learning perception.

Predictor	Unstandardized Coefficient (B)	Standard Error	Standardized Coefficient (β)	t	p
Constant	.665	.021	—	31.330	<.001
Platform_num	.003	.004	.005	.765	.444
cog_load	.035	.004	.058	8.307	<.001
Tool Fatigue ^*†*^	.673	.006	.768	111.953	<.001

*Note:*

*^a^Dependent variable: Learning Perception (higher scores indicate a more positive perception of the learning experience).*

*^b^All predictors are mean-centered; coefficients are unstandardized (B) and standardized (β).*

*^c^Model summary: R² = .598, F(3, 8612) = 4265.31, p < .001.*

*^†^Tool Fatigue is coded such that higher values represent greater fatigue (a negative construct); thus, the negative coefficient indicates that increased fatigue is associated with less positive learning perception.*

These quantitative findings underscore the psychological and experiential consequences of operating within a multi-platform ecosystem. To further unpack the mechanisms underlying these relationships—and to contextualize them within students’ lived experiences—the following analysis draws on the survey’s responses, structured around the five thematic domains outlined in the analytical framework. This mixed-methods approach enables a more nuanced understanding of how platform multiplicity manifests in practice and shapes learning outcomes.

### Theme 1: A crowded and diverse platform landscape

**Fig 4:**
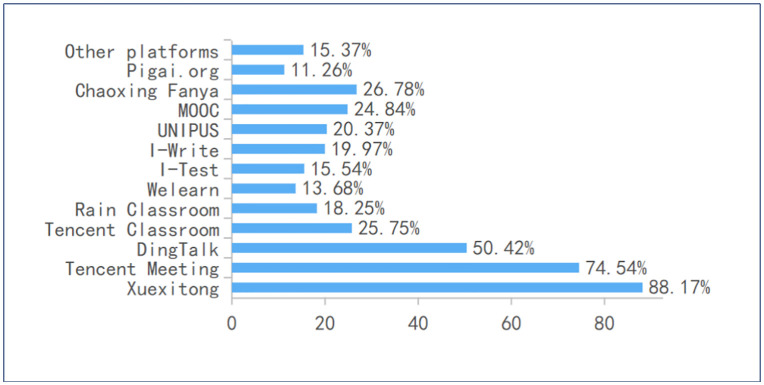
Online platforms use.

The findings reveal a highly fragmented digital learning environment in which students must navigate a crowded and heterogeneous platform ecosystem. Although a few systems—most notably Xuexitong (88.17%), Tencent Meeting (74.54%), and DingTalk (50.42%)—dominate usage (see Fig 4), students simultaneously engage with a wide array of additional LMS, communication, and MOOC platforms. This breadth of tools contributes to substantial platform proliferation: more than three‑quarters of respondents use at least three platforms for coursework, and nearly one‑quarter rely on five or more. Notably, this multiplicity is not confined to students taking multiple courses; even within a single subject such as English, over 40% report using three or more platforms. Collectively, these patterns indicate that platform multiplicity is a structural feature of students’ learning environments rather than an isolated or course‑specific phenomenon, suggesting a systemic diffusion of digital tools that may complicate students’ navigation and cognitive management of online learning tasks.

### Theme 2: The purpose and nature of platform engagement

The survey explored why and how students use these platforms, revealing a strong emphasis on administrative and assessment-related tasks.

**Fig 5:**
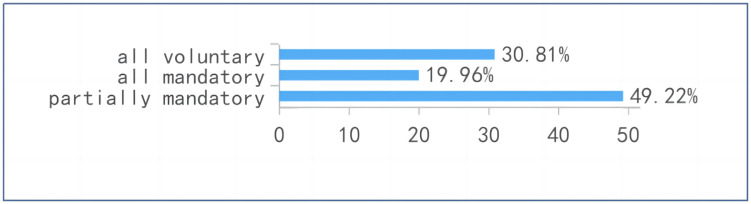
Mandatory vs voluntary use of platforms.

**Fig 6:**
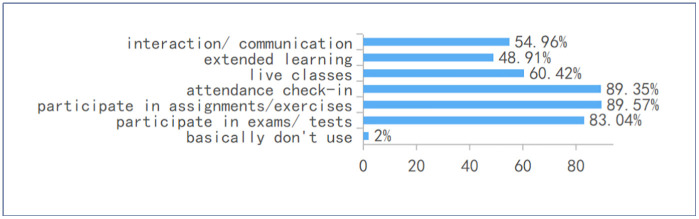
Primary purposes of use.

The findings indicate that students’ engagement with online platforms is shaped primarily by institutional requirements and task‑driven demands rather than by autonomous or exploratory learning practices. Nearly 70% of respondents reported that platform use was mandatory, underscoring the extent to which digital tools function as extensions of administrative control within the learning environment (see Fig 5). Consistent with this, the most frequently cited purposes—assignments (89.57%), attendance (89.35%), examinations (83.04%), and live classes (60.42%)—reflect highly instrumental and compliance‑oriented uses (see Fig 6), while activities associated with deeper learning, such as interaction or extended study, were comparatively less common. Moreover, students’ reported usage patterns show that platform engagement extends well beyond scheduled class time, with more than 70% indicating substantial after‑class use. Taken together, these patterns suggest that platforms operate less as pedagogical spaces for meaningful engagement and more as infrastructural mechanisms that structure, monitor, and regulate students’ academic responsibilities, thereby reinforcing an instrumental mode of digital participation.

### Theme 3: Perceived benefits and positive attitudes

Despite the challenges (detailed in the next section), students reported a range of benefits and expressed generally positive attitudes towards the platforms.

**Fig 7:**
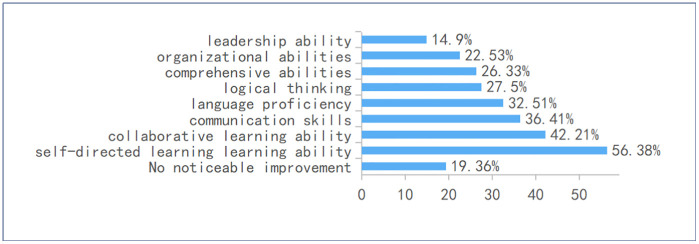
Skills or competencies improved through using online platforms.

Skill Development (Q14): Survey data reveals that students generally perceive online learning platforms as beneficial to their academic development, with a majority reporting improvements across multiple competencies. Most notably, 56.38% of respondents indicated that their self-directed learning ability had been enhanced, reflecting a perception that these platforms support autonomous study habits. As shown in Fig 7, collaborative learning skills were also positively impacted, with 42.21% of students acknowledging growth in this area, which students associated with the platforms’ potential to facilitate peer interaction. Communication skills saw improvement among 36.41% of participants, while 32.51% reported gains in language proficiency, whether in their native or foreign languages. Additionally, 27.5%, 26.33% and 22.53% of students noted enhancements in logical thinking, comprehensive abilities and organizational abilities respectively, suggesting a student perception that these tools may help foster broader cognitive skills. Importantly, only 19.36% of respondents felt that no competencies had been improved, underscoring a generally favorable attitude toward the educational value of these digital tools.

Positive Attitudinal Perceptions (Q15-Q20): Across a series of six Likert-scale questions, a strong majority of students agreed with positive statements about the platforms’ impact. For example, a substantial majority (74.81%) agreed that online platforms have facilitated interaction between students and teachers. Similarly, 71.02% expressed positive endorsement for the platforms’ role in enabling collaborative engagement. A combined 78.18% of respondents agreed that they habitually use online platforms to support their studies, and 77.31% expressed positive affirmation that the use of online platforms has promoted their learning. These findings suggest that students generally perceive online platforms as supportive of their academic experience. Taken together, these findings suggest that students generally perceive online platforms as supportive of peer engagement, their effectiveness in fostering meaningful teacher-student interaction remains less convincing, and the overall attitudinal trend reflects cautious optimism toward the communicative and developmental potential of digital learning environments.

### Theme 4: Significant challenges and drawbacks

Contrasting sharply with the positive attitudes is the high prevalence of reported problems and difficulties.

**Fig 8:**
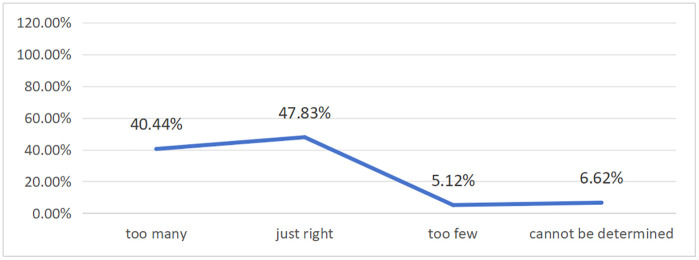
Students’ perception of the number of online platforms.

Perception of Quantity (Q8): A substantial portion of students feel overwhelmed by the number of platforms. 40.44% of respondents stated that the number of platforms they use is “too many”. While the largest group (47.83%) felt the number was “just right”, the fact that four in ten students feel burdened is a significant finding (see Fig 8). A Pearson correlation analysis revealed a significant positive correlation between the number of platforms used and our cognitive load measure (r = 0.169, p < 0.01) (see Table 3), supporting the hypothesis that as platform multiplicity increases, so does the level of perceived cognitive load.

**Table 3:** Pearson correlation between platform multiplicity and cognitive load.

Correlation			
		platform_num	cog_load
platform_num	Pearson correlation	1	.169**
	Sig.(2-tailed)		0
	N	8616	8616
cog_load	Pearson correlation	.169**	1
	Sig.(2-tailed)	0	
	N	8616	8616

** p < 0.01 (two-tailed)

**Fig 9:**
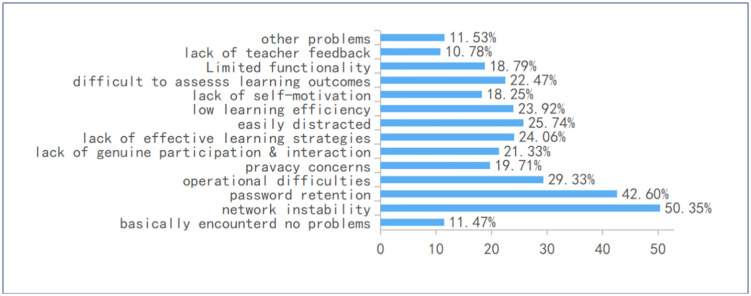
Problems or difficulties encountered during use.

Common Problems Encountered (Q13): Survey data from Question 13 (see Fig 9) reveals a diverse range of self-reported issues spanning technical, cognitive, pedagogical, and interpersonal domains. The most frequently reported problem was technical, with 50.35% of students citing network instability and platform crashes as significant barriers to effective engagement. Cognitive load also emerged as a major concern, with 42.60% of respondents struggling with password retention and 29.33% experiencing operational difficulties, suggesting that students perceive platform navigation itself as imposing a nontrivial cognitive burden. Pedagogical and personal challenges were also prominent: 25.74% of students reported being easily distracted, 23.92% experienced low learning efficiency, and 22.47% found it difficult to assess their learning outcomes. Despite generally favorable attitudes toward digital interaction, 21.33% of students identified a lack of genuine participation and interactivity as a persistent issue, suggesting a perception that platform-mediated communication may fall short of fostering meaningful engagement. Additionally, privacy concerns were noted by 19.71% of respondents. Notably, only 11.47% of students indicated that they had “basically encountered no problems”, underscoring the widespread and multifaceted nature of the challenges associated with online learning environments.

### Theme 5: Students’ perception of the impact of multiple online learning platforms (qualitative data)

The qualitative data from students’ open-ended reflections reveals a complex and ambivalent landscape regarding the use of multiple online learning platforms in higher education. While many respondents acknowledged the pedagogical benefits like accessibility, flexibility, and resource availability, they also expressed frustration with issues they linked to using multiple platforms.

On the positive side, many students appreciated the convenience of submitting assignments, accessing diverse learning resources, and reviewing course materials at their own pace. Several responses emphasized perceived improvements in self-directed learning and the ability to reinforce classroom content through playback and supplementary tools. These benefits suggest that online platforms, when effectively integrated, can support personalized and continuous learning.

However, the data also highlights significant challenges. A recurring concern was platform overload. This fragmentation was perceived as disruptive, which students associated with cognitive fatigue and reduced efficiency. Technical issues such as system crashes, slow performance, and forgotten passwords were commonly reported. Many students felt that platform use was overly formalized and lacked pedagogical depth, with limited feedback and superficial engagement.

Moreover, the psychological toll of digital learning was a prominent theme in student responses. Students described difficulties maintaining focus, declining motivation, and a sense of isolation. The constant exposure to notifications and non-academic content was seen as a distraction, which they felt undermined the potential benefits of online learning. While platforms offer structural advantages, their educational impact appears contingent on thoughtful design, integration, and instructional support.

## Discussion

**Fig 10:**
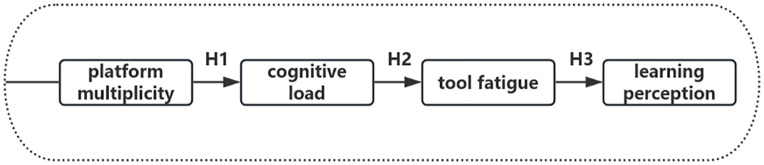
Proposed serial mediation model.

The results support a full serial mediation model (see Fig 10): platform multiplicity does not directly affect learning perception; rather, its influence is fully transmitted through a psychological pathway wherein increased platform usage is associated with heightened cognitive load, which in turn exacerbates tool fatigue, ultimately degrading students’ learning perceptions. Specifically, students using more platforms reported higher cognitive load (H1 supported), which subsequently intensified their sense of tool fatigue (H2 supported). Critically, this fatigue strongly undermined their perceived quality of learning (H3 supported). The non-significant direct effect in the full model (β = 0.005, p = .444) further indicates complete mediation.

The findings of this survey present a complex and often contradictory picture of the student experience with online learning platforms in Chinese higher education. While students express broad approval for the role of technology in their education, this positive sentiment coexists with significant, widespread reports of technical frustrations, cognitive overload, and pedagogical shortcomings. This discussion will interpret these findings by connecting them to the theoretical frameworks outlined earlier and explore the implications for various stakeholders.

### The paradox of perceived benefit and practical frustration

The most striking result is the dissonance between the high agreement rates on attitudinal questions (Theme 3) and the extensive list of problems reported (Theme 4). This can be described as the “necessary evil” [17] paradox: students appear to recognize the indispensable role of these platforms in the current educational system while simultaneously bearing the brunt of what they perceive as a flawed implementation.

This phenomenon is a clear manifestation of a core pedagogical tension: the gap between the theoretical promise of educational technology and the lived, practical experience of the student. Our data gives quantifiable evidence of this tension, showing that the same respondents simultaneously endorse the high pedagogical value and report high experiential difficulty. This suggests that the issue is not a simple rejection of technology, but rather a frustration stemming from a deeper misalignment between pedagogical intent and user experience, a finding that extends prior work on technostress [14].

This paradox can be partially explained by the Technology Acceptance Model (TAM). As platform use is mandatory for nearly 70% of students (Q9) and essential for core academic functions like submitting assignments and taking exams (Q10), the Perceived Usefulness is likely inflated by institutional compulsion. Students must find them useful to succeed. This high degree of perceived utility, driven by institutional requirement, likely overshadows the demonstrably low Perceived Ease of Use that arises from juggling multiple systems. Students may be expressing pragmatic acceptance relative to the alternative of being unable to complete their coursework, rather than authentic satisfaction with the quality of the digital learning experience. It may be that they have normalized this friction-filled digital environment as the new baseline for their academic life.

It is important to acknowledge that these findings are based on student self-reports, not objective measures of learning. However, from the students’ perspective, when a significant portion of their time and cognitive resources is reportedly consumed by navigating disparate platforms, managing passwords, and searching for scattered information, they perceive that the mental bandwidth available for actual learning is reduced. Consistent with this, students frequently described their experiences using terms such as “low efficiency”, “tedious”, and “time-wasting”. More critically, the percieved cognitive overload associated with platform fragmentation appears to encourage surface-level engagement: many resort to what they call “brushing classes” (i.e., mechanically completing required clicks or tasks) merely to fulfill course requirements, rather than engaging deeply with the subject matter. One student succinctly summarizes the futility: “Too many software is to complete brushing classes, no practical significance.”

### Cognitive overload in practice: From theory to student reality

The survey data provides compelling evidence for interpreting the student experience through the lens of Cognitive Load Theory (CLT). The challenges cited by students, such as forgetting passwords, operational difficulty/forgetting URLs, and the general feeling of using “too many” platforms, are consistent with manifestations of extraneous cognitive load. While we did not use a standalone, validated scale for cognitive load in the descriptive part of the survey, these self-reported issues serve as practical indicators of the cognitive burdens students perceive. Our inferential analysis further supports this interpretation. According to CLT, the mental energy a student expends on such platform-management tasks is energy diverted away from the primary learning goal. This diversion of cognitive resources from the intrinsic (subject complexity) and germane (knowledge construction) loads to the extraneous (interface management) load is what students appear to be describing.

The fact that over 77% of students use three or more platforms suggests that this is not an isolated issue but a systemic one. The burden of technological proliferation often leads to what is called tool fatigue, which refers to the mental and physical exhaustion resulting from the constant need to manage, switch between, and operate numerous digital applications. The student responses provide overwhelming evidence of this phenomenon. A dominant and recurring theme is the sheer volume of required software and apps. Students repeatedly lament, “Too many software”, “Too many apps”, “Platform too many, memory not enough”, and “App platform too many, often appear confused”.

This fragmentation forces students into a cycle of constant app-switching, which they find to be not only time-consuming but also deeply fatiguing. Each platform often has its own unique interface and navigation logic, requiring students to re-learn basic operations repeatedly. Each additional platform adds a layer of extraneous cognitive load, inadvertently creating a fragmented experience that taxes students’ limited working memory before any real learning has even begun. This friction transforms the learning process from a seamless experience into a series of frustrating, disjointed tasks, leading to a sense of being overwhelmed and demotivated.

### Instrumental engagement vs. deep learning

The data on the purpose of platform use (Q10) is particularly revealing. The top three uses, namely, assignments, attendance, and tests, are primarily administrative and evaluative. This pattern of use positions the platforms as tools of academic management and surveillance rather than as environments for intellectual exploration. In contrast, extended learning and interaction/communication lag behind.

This suggests that platforms are being used in a highly instrumental way. They are the digital hoops through which students must jump to get their grades. This finding challenges the top-reported benefit of “improved autonomous learning ability”. Is this true autonomy—the self-motivated pursuit of knowledge—or is it merely the “autonomy” to complete mandated tasks on one’s own schedule? The data suggests the latter. Students are learning to be self-sufficient in managing a complex administrative workload, which is a skill in itself, but it may not be the deep, self-directed learning that educators hope to foster. This again highlights the pedagogical tension: while platforms are intended to foster “autonomous learning”, students report that the lack of a streamlined, user-friendly experience hinders true autonomy. They spend more time managing the tools than directing their own learning. Comments such as “poor self-discipline” and “can’t control usage time” suggest that platform multiplicity may be percieved as a hindrance to, rather than a support for learner autonomy.

Beyond technical and instructional concerns, students reported psychological strain associated with platform use. A recurring theme was diminished self-regulation, with reflections such as “poor self-control”, “easily distracted”, and “decline in motivation”. The constant exposure to notifications, advertisements, and non-academic content was seen as a source of cognitive interference, leading some students to describe the platforms as a double-edged sword capable of facilitating learning but equally prone to undermining focus and discipline. This tension was particularly pronounced among students who felt that platform use was externally imposed, indicating a disconnect between institutional expectations and student engagement.

### The quantity vs. quality of interaction

The findings on interaction also reveal a potential disconnect. A high percentage of students agree that platforms promote both teacher-student and student-student interaction. However, a notable 21.3% of students simultaneously identify a “lack of real participation and interactivity” as a key problem (Q13).

This suggests a difference between the perceived quantity and quality of interaction. Platforms make it easy to generate interaction events — a click on a “like” button, a one-sentence reply to a forum post. This increases the measurable volume of interaction. However, as suggested by student comments, these low-effort interactions may not contribute to a robust Community of Inquiry (CoI). They may lack the social presence and sustained discourse necessary for building cognitive presence. The feeling of “unreal” participation likely stems from interactions that are perceived as superficial or performed solely for credit. The fragmentation across platforms can further dilute the sense of community, as discussions happening in a DingTalk group are disconnected from content on Xuexitong.

Pedagogically, students critiqued the superficiality of interaction and the lack of meaningful feedback. Student comments suggest that many platforms fail to replicate the depth of engagement found in traditional classroom settings. Some students also noted that platform features, such as automated grading or rigid assignment formats, were “mechanical” and “lacked substantive educational value”, reinforcing the perception that digital tools are often used for administrative compliance rather than pedagogical enrichment.

### Implications for stakeholders

While online learning platforms hold immense potential, the current implementation—characterized by a fragmented, multi-app ecosystem—is counterproductive. The resulting tool fatigue and cognitive overload act as significant barriers to effective learning. Students are not resisting technology; they are resisting a poorly designed, inefficient system that prioritizes administrative convenience over pedagogical effectiveness and user experience. The findings of this study have clear implications for the key actors in the higher education ecosystem.

For **Educational Institutions**: The most urgent need is to develop a coherent institutional digital strategy. Allowing a “digital wild west” where every instructor or department chooses their own set of tools is demonstrably burdensome for students.

Institutions should consider:

- Standardization and Integration: Selecting a core, reliable LMS and ensuring that any additional required tools can be seamlessly integrated through standards like Learning Tools Interoperability (LTI) or Single Sign-On (SSO) to reduce the cognitive load of managing multiple logins.
- Technical Support and Infrastructure: The top problem cited was network instability. Institutions must invest in robust IT infrastructure and responsive technical support to ensure the basic functionality of their chosen platforms.

For **Instructors**: The responsibility for effective pedagogical use of technology falls heavily on instructors. They should:

- Practice “Digital Minimalism”: Be mindful of the cumulative burden on students. Before adding another platform to a course, instructors should ask if its function can be accomplished within the existing toolset and if its pedagogical benefit truly outweighs the added cognitive load.
- Design for Deep Engagement: Move beyond using platforms for attendance and simple quizzes. Instructors need training and support to design activities that foster critical thinking, meaningful collaboration, and a genuine sense of community, focusing on the quality, not just the quantity, of interaction.

For **Platform Developers**: The feedback from this large user base is invaluable. Developers should prioritize:

- User Experience (UX) and Reliability: The high incidence of forgotten passwords and operational difficulties points to a need for more intuitive design. Above all, platform stability is paramount.
- Interoperability: Designing platforms with open APIs and adherence to educational standards (like LTI) will allow institutions to build integrated ecosystems, which would directly address the core problem identified in this study.
- Meaningful Analytics: Provide dashboards that help instructors and students assess the quality of learning and engagement, not just the number of clicks and posts.

## Conclusion

This study set out to analyze the benefits and drawbacks of using multiple online learning platforms in higher education, based on a large-scale survey of 8,616 Chinese university students. The findings reveal an educational landscape saturated with digital tools, where the average student must navigate a complex ecosystem of three or more platforms. The central conclusion of this analysis is the existence of a profound tension: while students conceptually approve of these platforms and recognize their necessity, their daily experience is marred by significant technical challenges, administrative burdens, and a cognitive overload that detracts from the primary goal of learning.

The research demonstrates that platforms are predominantly used for instrumental purposes—managing assignments, tracking attendance, and conducting exams—rather than as spaces for deep intellectual inquiry and community building. This instrumentalization, coupled with the friction of a fragmented digital environment, risks turning powerful tools for learning into mere instruments of academic administration. The “autonomous learning” that students feel they are gaining may be more about managing a complex technological workload than about genuine intellectual self-direction.

The findings underscore that the path forward is not simply to add more technology, but to be more thoughtful about its implementation. The challenge for higher education is to move from an era of platform proliferation to an era of platform integration. This requires a concerted effort from all stakeholders. Institutions must lead with strategic vision, investing in integrated systems and robust infrastructure. Instructors must receive the pedagogical training needed to transform these tools from content-delivery mechanisms into vibrant learning communities. And developers must prioritize user experience, reliability, and interoperability to reduce the extraneous cognitive load currently placed on students.

Future research should build upon these findings through qualitative studies to explore the “why” behind the reported frustrations and satisfactions. Longitudinal research could track how student perceptions and coping strategies evolve over their university careers. Ultimately, the goal must be to create a seamless, supportive, and engaging digital learning environment where technology serves pedagogy, not the other way around, and where the focus can return from managing the tools to the timeless pursuit of knowledge.

### Limitations

This study has several limitations. First, as a cross-sectional study, it cannot capture changes in student perceptions over time. A longitudinal study might reveal how attitudes and challenges evolve as students progress through their academic careers. Second, the heavy concentration of freshmen and sophomores (over 91% of the sample) means the findings may not be fully generalizable to senior undergraduate students, whose cumulative experience with educational technology might be different. Despite these limitations, the large sample size and the breadth of the questionnaire provide a robust and valuable foundation for understanding the current state of multi-platform usage in Chinese higher education.

## Abbreviations

CoL: Community of Inquiry
TAM: Technology Acceptance Model
CLT: Cognitive Load Theory
LMS: Learning Management System.
